# Early Adoption of Technically Complex Robotic Ventral Hernia Repair in a Low-Volume Center: A Retrospective Comparative Analysis with Consecutive Open Cases

**DOI:** 10.3390/jcm15176778

**Published:** 2026-09-01

**Authors:** Andraž Hubad, Aleš Tomažič, Blaž Trotovšek, Primož Sever, Jan Grosek

**Affiliations:** 1 Department of Abdominal Surgery, University Medical Centre Ljubljana, Zaloška cesta 7, 1000 Ljubljana, Slovenia; 2 Faculty of Medicine, University of Ljubljana, Korytkova 2, 1000 Ljubljana, Slovenia

**Keywords:** robotic surgery, ventral hernia, incisional hernia, abdominal wall reconstruction, transversus abdominis release (TAR), learning curve, low-volume center, retromuscular repair, minimally invasive surgery

## Abstract

**Background/Objectives**: Robotic-assisted abdominal wall reconstruction is increasingly used for ventral hernias; however, implementation in low-volume settings and early adoption of technically complex cases remain controversial. This study aimed to evaluate perioperative outcomes of robotic ventral hernia repair with early inclusion of technically complex cases and to compare these results with open surgery. **Methods**: Prospectively collected data were retrospectively analyzed for consecutive technically complex robotic ventral hernia repairs performed between September 2021 and December 2025. A cohort of consecutive open Rives–Stoppa repairs was included for comparison (*n* = 21 per group). Outcomes included operative time, length of hospital stay (LOS), and perioperative complications. **Results**: Operative time was significantly longer in the robotic group (median 310 vs. 118 min, *p* < 0.001). The median LOS was significantly shorter following robotic repair (3 vs. 5 days, *p* = 0.0018). Postoperative complications occurred in 10% of robotic cases and 24% of open cases (*p* = 0.41). Major complications and reoperation rates were low and comparable between groups. Technically complex robotic cases, including procedures requiring component separation, were introduced early and were associated with acceptable short-term outcomes. **Conclusions**: Technically complex robotic hernia repair was associated with significantly shorter hospital stay compared with open surgery, despite longer operative times and higher procedural complexity. Complication rates were comparable between groups. Early inclusion of technically complex cases was not associated with a statistically significant increase in short-term complications.

## 1. Introduction

Incisional hernias represent a frequent and clinically relevant problem in abdominal wall surgery. Open retromuscular repair, most commonly performed using the Rives–Stoppa technique, is widely regarded as a reference standard for midline incisional hernias and has been widely utilized with well-proven outcomes [1]. Current European Hernia Society (EHS) guidelines recommend mesh placement in the retromuscular plane, while intraperitoneal mesh placement should be used with caution due to potential long-term complications [2].

Over recent decades, minimally invasive approaches have been increasingly adopted in ventral hernia repair. Laparoscopic techniques have demonstrated advantages in terms of reduced wound complications and shorter recovery; however, their applicability in complex hernia cases has historically been limited. In particular, laparoscopic repair has frequently been associated with intraperitoneal mesh placement and may be limited in achieving primary fascial closure and restoration of abdominal wall anatomy, particularly in larger defects; moreover, these techniques are associated with a recognized learning curve [3,4].

Robotic-assisted surgery addresses several of these limitations by combining enhanced three-dimensional visualization, improved instrument articulation, and precise intracorporeal suturing [5,6]. These features enable access to retromuscular and preperitoneal planes, facilitating fascial closure, anatomical abdominal wall reconstruction, and extraperitoneal mesh placement in accordance with current guidelines [2,5,6]. As a result, advanced reconstructive techniques, including transversus abdominis release (TAR) and other component separation techniques, have increasingly been performed in a minimally invasive robotic setting [5,7].

Although some studies have reported overcoming of the learning curve after as few as 7–12 cases in simple hernia procedures, particularly robotic transabdominal preperitoneal (TAPP) repair [8,9], more recent evidence suggests considerable variability in the reported learning curve for robotic TAPP. A 2026 scoping review of CUSUM-based studies reported operative-time stabilization points ranging from 12 to 138 procedures, with a median of 32 cases, and estimated that approximately 25–35 procedures may be compatible with operative-time stabilization in structured training settings. The authors emphasized that this estimate is hypothesis-generating rather than a credentialing benchmark [10]. In robotic Rives–Stoppa repair, operative time was reported to decrease after approximately 29 cases, while adverse outcomes decreased after approximately 51 cases [11]. In robotic TAR, the learning curve was reported to be overcome between 49 and 75 cases [12]. These findings suggest that learning-curve estimates should be interpreted in relation to the specific procedure and case complexity rather than as a universal threshold for robotic proficiency.

The European Hernia Society (EHS) training pathway recommends a structured, stepwise approach to robotic abdominal wall surgery, with progressive escalation from less complex to more advanced procedures during the learning phase [13]. This approach has also been evaluated clinically. Latif et al. reported low postoperative morbidity and satisfactory short-term outcomes during implementation of the pathway, progressing from robotic TAPP to TARUP/eTEP and subsequently to TAR [14]. Similarly, Anton et al. reported favorable early outcomes following structured implementation of robotic TARUP in a low-volume community hospital, although their initial cohort was restricted to selected primary midline W1 hernias [15]. However, the safety and feasibility of introducing more technically complex robotic ventral hernia procedures relatively early during program implementation remain less well described.

Strict adherence to such structured training pathways may not be feasible in all clinical settings. Limited access to robotic platforms, competition between surgical specialties for operating time, and institutional resource constraints may influence the implementation of robotic programs, particularly in low-volume centers.

In addition, economic considerations remain a major factor in the adoption of robotic hernia surgery. Notably, a single-center study even reported that robotic TAPP may be less expensive than laparoscopic repair, with cost differences of approximately USD 100–200 per case [16]. However, higher costs have been reported for robotic repair, particularly for routine inguinal hernia procedures [17,18]. Whether this cost difference remains equally relevant for more complex abdominal wall procedures is less clear, as recent data suggest that the relative economic impact of robotic surgery may vary according to hernia type and procedural complexity [19].

The term “complex ventral hernia” is used in the literature, although no single universally accepted definition or validated threshold for complexity has been established. The EHS Delphi consensus identified multiple criteria contributing to the complexity of incisional hernias, reflecting the multifactorial nature of the concept [20,21,22,23].

For the purposes of robotic surgery, however, the complexity of the procedure itself is also relevant. The EHS robotic abdominal wall surgery pathway recognizes progressive escalation from less complex procedures toward more advanced reconstructive procedures, including retromuscular repair and component separation procedures such as transversus abdominis release [13]. In the present study, the term “technically complex robotic ventral hernia repair” is therefore used to describe the procedural complexity of the robotic reconstruction.

In our institution, robotic abdominal wall surgery was introduced under specific organizational constraints. Implementation of a robotic hernia program preceded the publication of the EHS robotic abdominal wall surgery training pathway and was characterized by limited access to the robotic platform, largely due to prioritization of other surgical domains, particularly oncologic procedures. As a result, overall procedural volume remained low, while the use of robotic systems for less technically complex cases was limited. Consequently, more technically complex abdominal wall reconstructions were introduced relatively early in the implementation phase.

We hypothesized that under specific institutional conditions, early implementation of technically complex robotic ventral hernia repair could be safely performed despite limited institutional experience.

## 2. Materials and Methods

### 2.1. Study Design and Patient Selection

Prospectively collected data were retrospectively analyzed for all consecutive patients undergoing robotic hernia repair at the University Medical Centre Ljubljana between September 2021 and December 2025. Of the 44 robotic procedures performed during this period, 21 technically complex robotic ventral hernia repairs were included in the analysis. The first technically complex robotic case, a transversus abdominis release (TAR), was performed in May 2022 as the 8th robotic hernia procedure overall.

The allocation of patients to either robotic or open repair was determined primarily by surgeon preference and institutional availability of the robotic platform, rather than by a formal protocol or randomization. Patients were considered for robotic repair when the robotic platform was available and when the surgeon deemed the approach technically appropriate based on hernia characteristics. Open repair was performed in consecutive patients scheduled for elective ventral hernia repair through the standard surgical waiting list. No formal contraindications to robotic repair were defined beyond general anesthetic risk. Patient preference did not play a role in the allocation of surgical approach.

For comparison, consecutive patients undergoing open ventral hernia repair using the Rives–Stoppa technique were included. The Rives–Stoppa technique was selected as the open comparator and represents a relatively standardized procedure performed routinely by multiple surgeons at the institution. The number of open cases was determined by the number of technically complex robotic cases, resulting in a 1:1 ratio between the two groups. Inclusion of the open cohort began at the start of 2023, with consecutive eligible cases collected until 21 patients were identified; the last included open case was operated on in September 2024. One patient in whom adjunctive fascial traction (Fasciotens^®^) was used was excluded from the open group. The final analysis included 21 patients in each group.

### 2.2. Surgical Technique and Case Stratification

All robotic procedures were performed by a single surgeon with extensive prior experience in robotic-assisted colorectal surgery (>100 procedures) using the da Vinci Xi platform.

Robotic cases were stratified according to procedural complexity into three categories: straightforward, intermediate, and technically complex. Straightforward cases included primary inguinal and small umbilical hernias. Intermediate cases comprised umbilical hernias with rectus diastasis, selected recurrent inguinal hernias, and simple parastomal repairs. Technically complex cases included midline incisional hernias, lateral hernias, parastomal hernias with concurrent midline hernia, and cases requiring component separation techniques. This classification was used solely to define study eligibility and does not represent a validated definition of complex ventral hernia. Only patients in the technically complex category were included in the comparative analysis with the open group. Straightforward and intermediate robotic procedures were excluded from the comparative analysis.

### 2.3. Data Collection and Definitions

The following variables were collected: patient age at surgery, sex, body mass index (BMI), American Society of Anesthesiologists (ASA) score, number of previous hernia repairs, presence of recurrent hernia, hernia type and location, and presence of stoma.

Operative variables included operative time (skin-to-skin), intraoperative complications, and need for conversion.

Length of hospital stay (LOS) was defined as the number of postoperative days, calculated from the date of surgery to the date of discharge, with same-day discharge recorded as one day.

Postoperative outcomes included any postoperative complication, major complications defined as Clavien–Dindo grade ≥ III, reoperation, and readmission within 30 days. Follow-up data regarding long-term recurrence were not systematically available and were therefore not included in the analysis.

### 2.4. Outcomes

The primary outcome of the study was postoperative length of hospital stay (LOS). Secondary outcomes included operative time, any postoperative complication, major complications (Clavien–Dindo ≥ III), reoperation rate, and readmission rate within 30 days.

### 2.5. Statistical Analysis

Continuous variables were expressed as median and interquartile range (IQR) and compared using the Mann–Whitney U test. Categorical variables were compared using Fisher’s exact test.

A *p*-value < 0.05 was considered statistically significant. Statistical analysis was performed using R software (version 4.6.0; R Foundation for Statistical Computing, Vienna, Austria).

### 2.6. Use of Generative Artificial Intelligence

During the preparation of this manuscript, ChatGPT (OpenAI, GPT-5.5) was used for sentence formulation and language refinement. The tool was not used for data collection, data analysis, statistical analysis, interpretation of results, or generation of scientific content. All AI-assisted text was reviewed and edited by the authors, who remain fully responsible for the content of the manuscript.

## 3. Results

### 3.1. Patient Characteristics

A total of 42 patients were included in the comparative analysis, comprising 21 technically complex robotic cases and 21 open cases.

The technically complex robotic cohort comprised 21 procedures: 2 unilateral transversus abdominis releases (TAR), 3 robotic Pauli repairs, 4 retromuscular Rives–Stoppa repairs, 1 robotic Rives–Stoppa repair combined with recurrent inguinal hernia repair, 9 preperitoneal lateral hernia repairs performed with the patient in the lateral decubitus position, and 2 preperitoneal midline mesh repairs. All meshes were placed in extraperitoneal positions, and all meshes measured at least 14 cm in the shorter dimension (width).

Baseline patient characteristics, summarized in Table 1, were comparable between the two groups. Patient age (median 59 vs. 59 years; IQR 49–69 vs. 52–71), body mass index (BMI) (median 28.1 vs. 29.0 kg/m^2^; IQR 25.4–30.9 vs. 26.8–32.3), and sex distribution (male 62% vs. 67%) were similar between groups. The majority of patients in both groups were classified as ASA II–III.

### 3.2. Operative Details

Operative time was significantly longer in the robotic group compared with the open group (median 310 vs. 118 min, *p* < 0.001). The interquartile range (IQR) was 257–390 min in the robotic group and 86–133 min in the open group. The mean operative time was 331 min (range 141–575) in the robotic group and 124 min (range 63–236) in the open group. Component separation techniques were performed in 5 patients (24%) in the robotic group and in none of the open cases. A stoma was present in 3 patients (14%) in the robotic group and in 0 patients in the open group. Recurrent hernia was present in 7 patients (33.3%) in the robotic group and 6 patients (28.6%) in the open group (*p* = 1.000); among these, 3 patients in the robotic group had undergone 2 previous repairs and 4 had undergone 1 previous repair, compared to 2 and 4 patients in the open group, respectively.

**Figure 1:**
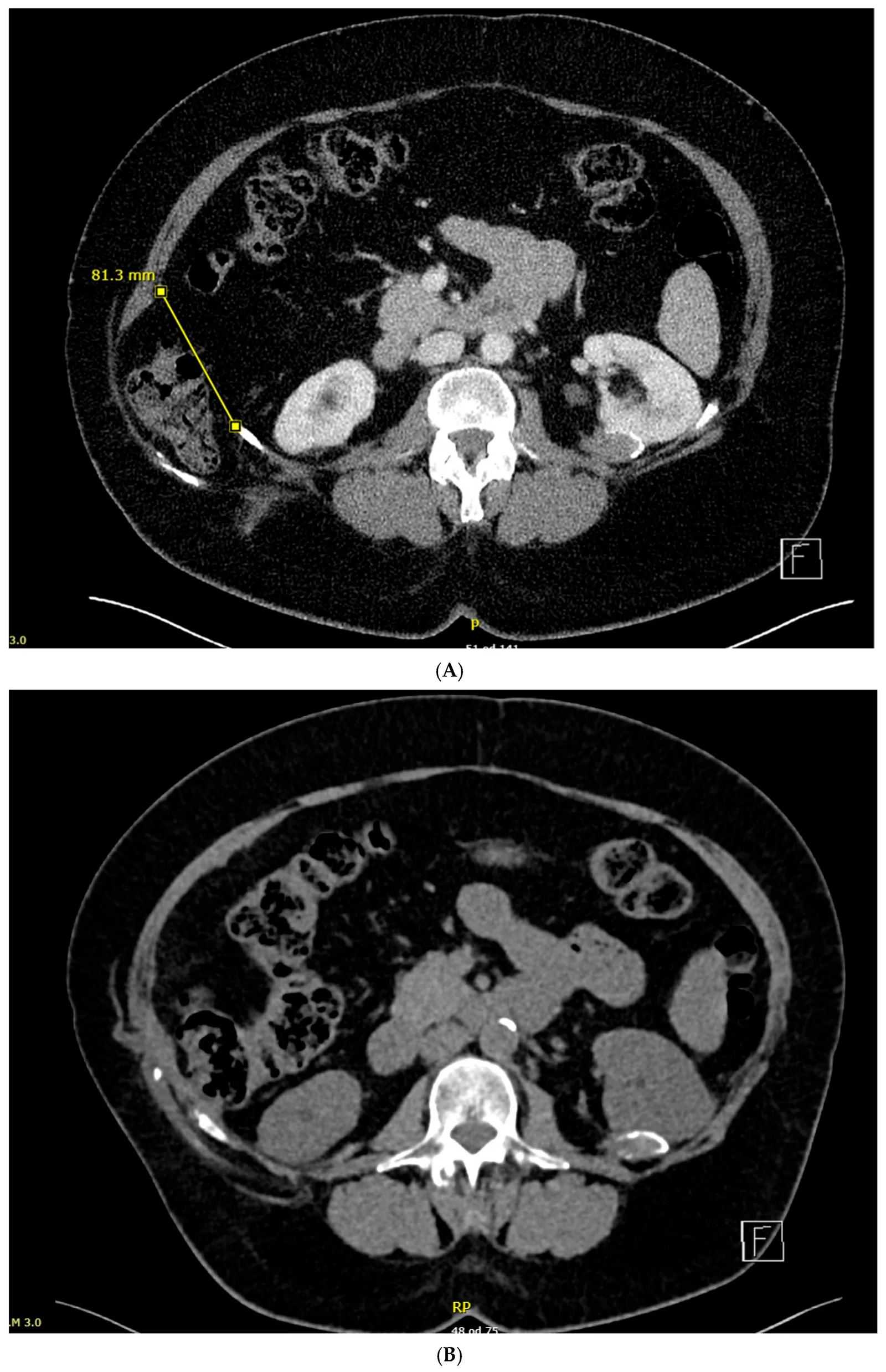
Representative computed tomography images of a recurrent lateral incisional hernia following lumbotomy for partial nephrectomy. (**A**) Preoperative axial CT demonstrating a recurrent lateral hernia after previous failed onlay hernioplasty. (**B**) Postoperative axial CT at 5 months following robotic preperitoneal repair performed with the patient in the lateral decubitus position, demonstrating successful abdominal wall reconstruction with mesh in the extraperitoneal position.

Intraoperative complications occurred in 3 patients (14%) in the robotic group and 4 patients (19%) in the open group (*p* = 1.000). In the robotic group, one complication involved injury to an ileal conduit and two involved serosal injury of the small bowel. In the open group, all intraoperative complications consisted of serosal injury of the small bowel requiring suture repair. No conversions to open surgery were observed in the robotic group. A representative case is illustrated in Figure 1, demonstrating a recurrent (onlay hernioplasty) lateral incisional hernia following lumbotomy for partial nephrectomy, with preoperative and postoperative imaging.

### 3.3. Postoperative Outcomes

The median length of hospital stay was significantly shorter in the robotic group compared with the open group (median 3 vs. 5 days, *p* = 0.0018). The interquartile range (IQR) for length of hospital stay was 2–4 days in the robotic group and 3–8 days in the open group.

Postoperative complications occurred in 2 patients (10%) in the robotic group and 5 patients (24%) in the open group, with no statistically significant difference between groups (*p* = 0.41). In the robotic group, postoperative complications consisted of a seroma requiring reoperation and a complication involving the ileal conduit. In the open group, complications included a mesh-related surgical-site infection requiring reoperation, cephalic vein thrombosis treated with anticoagulation, postoperative ileus, a febrile inflammatory episode treated empirically with antibiotics, and purulent tonsillitis.

Major complications (Clavien–Dindo ≥ III) were rare, with one event recorded in each group (5% vs. 5%, *p* = 1.000).

Reoperation was required in one patient in each group (5% vs. 5%, *p* = 1.000). Readmission occurred in two patients (10%) in the open group and none in the robotic group (*p* = 0.49).

Operative and postoperative outcomes are summarized in Table 2.

### 3.4. Progression of Procedural Complexity in Robotic Hernia Cases

**Figure 2:**
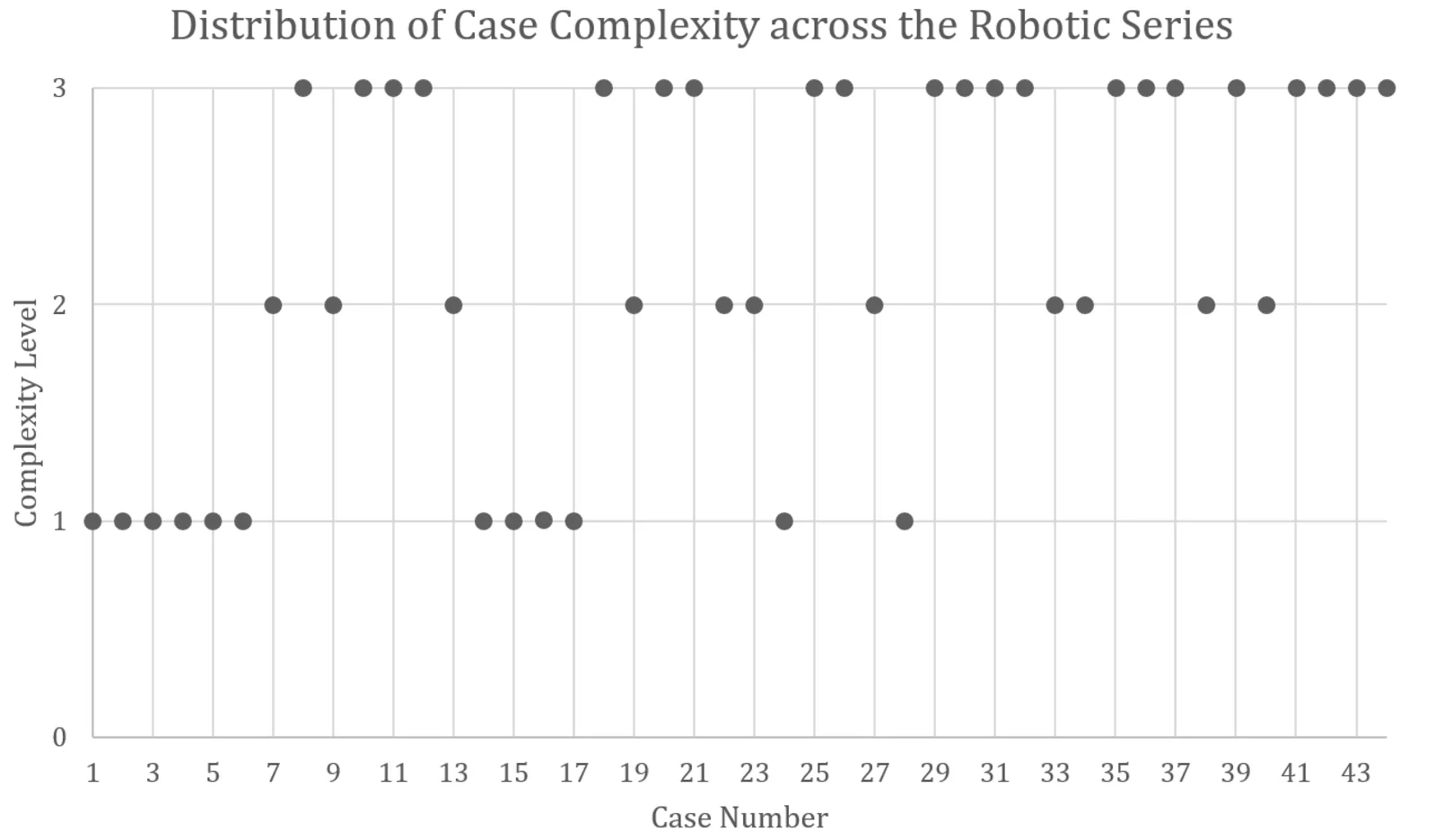
Distribution of case complexity across the robotic series. Complexity levels were defined as 1 (straightforward), 2 (intermediate), and 3 (technically complex).

Robotic procedures included a spectrum of case complexity. Technically complex robotic procedures were introduced early during the implementation of the robotic hernia program. These included component separation cases as early as cases 8, 10, 11, and 12, demonstrating early adoption of advanced operative techniques within the robotic program (Figure 2).

## 4. Discussion

The aim of this study was not to advocate early adoption of technically complex robotic hernia repair outside a structured training pathway, but rather to report the outcomes of such an implementation under specific institutional real-world conditions. In this cohort, technically complex robotic procedures were associated with a shorter length of hospital stay and longer operative time compared with open repair, while no statistically significant difference was observed in postoperative complication rates.

Operative time in the technically complex robotic group was significantly longer than in the open group (median 310 vs. 118 min, *p* < 0.001). This likely reflects both the early phase of robotic program implementation and the greater procedural complexity of the technically complex robotic group compared with the open group. The robotic cohort included technically complex abdominal wall reconstructions and cases requiring component separation techniques, which were introduced early in the robotic experience.

These findings should also be considered in the context of published experience with structured robotic training. Latif et al. reported favorable short-term outcomes following implementation of the European Hernia Society robotic abdominal wall surgery pathway, with progressive introduction of procedures from robotic TAPP to TARUP/eTEP and subsequently to TAR. In their series, median operative time for robotic TAR was 426 min, compared with 167 min for TARUP/eTEP procedures, highlighting the longer operative times associated with the more advanced TAR procedure [14]. Similarly, previous learning-curve studies have demonstrated progressive reductions in operative time with increasing experience, with approximately 29 cases required before operative-time improvement was observed for robotic Rives–Stoppa repair [11]. Taken together, these data support a progressive training model and suggest that the operative times observed in our early experience may reflect the expected effects of limited experience and case complexity rather than an intrinsic limitation of the robotic approach.

Despite this, we observed a significantly shorter length of hospital stay in the robotic group, which aligns with previously reported benefits of minimally invasive abdominal wall reconstruction [24,25]. Importantly, this reduction in hospital stay was observed in our study despite the higher procedural complexity of the robotic group, including five component separation procedures compared with none in the open group. This finding is notable in the context of the different case mix between the groups, although this difference limits direct comparison of postoperative outcomes. This observation is consistent with previous comparative studies and meta-analyses reporting shorter hospital stays with robotic approaches to complex abdominal wall reconstruction despite longer operative times [24,25,26].

With regard to complications, no statistically significant difference was observed between groups. In the robotic group, one patient required reoperation for a postoperative seroma that was initially suspected to represent a mesh infection. Computed tomography findings demonstrated fluid collection with gas inclusions raising suspicion for abscess formation. However, intraoperative findings revealed no evidence of infection, and microbiological samples remained sterile. The intraoperative findings were consistent with seromatous fluid, and the hernia repair remained intact; simple excision of the hernia sac was performed, followed by an uneventful postoperative course.

In one patient, robotic repair of a recurrent hernia was performed in conjunction with urological metastasectomy for clear cell renal carcinoma, allowing both procedures to be completed during a single operation.

In the open group, one patient required multiple reoperations due to mesh infection. Previous systematic reviews and meta-analyses have reported lower surgical-site infection rates with robotic approaches compared with open ventral hernia repair, including in analyses specifically evaluating robotic versus open TAR [25,26]. In our cohort, however, this difference was not statistically demonstrable, likely reflecting the small sample size and substantial difference in case complexity between groups. Furthermore, despite longer operative times and greater procedural complexity of the robotic group, including lateral hernias requiring positioning in the lateral decubitus position, and component separation procedures, no statistically significant difference in overall postoperative complication rates was observed between groups. These findings should be interpreted cautiously given the small sample size and the differences in case complexity between the groups.

This study has several limitations. The relatively small sample size limits statistical power, particularly for infrequent outcomes such as major complications and readmissions. As such, the present study primarily reflects institutional experience rather than providing definitive evidence for broader clinical conclusions. The retrospective design introduces potential selection bias, and long-term follow-up data, including recurrence rates, were not systematically available. The specific institutional circumstances under which the robotic program was implemented may also limit the generalizability of these findings to centers with different access to robotic platforms, case volumes, or training structures.

A particularly important limitation of this study is the inherent difference in case complexity between the two groups, which represents a significant source of selection bias. The robotic group included technically complex procedures such as transversus abdominis release (TAR), lateral hernia repairs requiring lateral decubitus positioning, and parastomal hernias with concurrent midline defects, none of which were present in the open group. This imbalance means that direct comparison of operative time and postoperative outcomes between groups must be interpreted with caution. The longer operative times observed in the robotic group likely reflect both the early phase of program implementation and the higher procedural complexity, rather than an inherent disadvantage of the robotic approach. The non-significantly different complication rates, while encouraging, should likewise be interpreted with caution, as the higher procedural burden in the robotic group may have obscured true differences between approaches, and no definitive conclusions regarding the relative safety of either technique can be drawn from these data.

While adherence to structured training pathways may have further optimized outcomes, such pathways were not fully feasible in our setting. Nevertheless, the approach adopted allowed us to offer selected patients extraperitoneal mesh placement and abdominal wall reconstruction using minimally invasive techniques. Within the limitations of this analysis, these procedures were associated with acceptable short-term outcomes, although the findings should not be interpreted as evidence that structured training pathways can be safely bypassed.

## 5. Conclusions

In this single-center experience, early implementation of technically complex robotic procedures was associated with a significantly shorter hospital stay compared with the open group, despite longer operative times and greater procedural complexity. Complication rates were comparable between groups. Early inclusion of technically complex cases, including procedures requiring component separation, was not associated with a statistically significant increase in short-term complications. In this specific institutional setting, early implementation of technically complex robotic procedures was feasible. However, given the limited sample size and single-center design, definitive conclusions cannot be drawn. This study does not challenge the recommendation that a structured, stepwise approach as outlined by current training pathways remains the preferred standard.

## Figures and Tables

**Table 1:** Baseline characteristics.

Variable	Robotic (*n* = 21)	Open (*n* = 21)	*p*-Value
Age (years)	59 (49–69)	59 (52–71)	0.67
BMI (kg/m^2^)	28.1 (25.4–30.9)	29.0 (26.8–32.3)	0.35
Sex (male)	13 (62%)	14 (67%)	1.00
Sex (female)	8 (38%)	7 (33%)	—
ASA I	3 (14%)	0 (0%)	
ASA II	12 (57%)	10 (48%)	
ASA III	6 (29%)	11 (52%)	0.12

**Table 2:** Operative and postoperative outcomes.

Outcome	Robotic (*n* = 21)	Open (*n* = 21)	*p*-Value
Operative time (min)	310 (257–390)	118 (86–133)	<0.001
Length of stay (days)	3 (2–4)	5 (3–8)	0.0018
Intraoperative complications	3 (14%)	4 (19%)	1.000
Any postoperative complication	2 (10%)	5 (24%)	0.41
Clavien–Dindo ≥ III	1 (5%)	1 (5%)	1.000
Reoperation	1 (5%)	1 (5%)	1.000
Readmission	0 (0%)	2 (10%)	0.49

Data are presented as median (IQR) or number (%). *p*-values were calculated using the Mann–Whitney U test for continuous variables and Fisher’s exact test for categorical variables.

## Data Availability

The data presented in this study are available on request from the corresponding author due to privacy restrictions.

## Abbreviations

The following abbreviations are used in this manuscript:

TAR	Transversus abdominis release
TAPP	Transabdominal preperitoneal repair
LOS	Length of hospital stay
BMI	Body mass index
ASA	American Society of Anesthesiologists
IQR	Interquartile range
EHS	European Hernia Society
